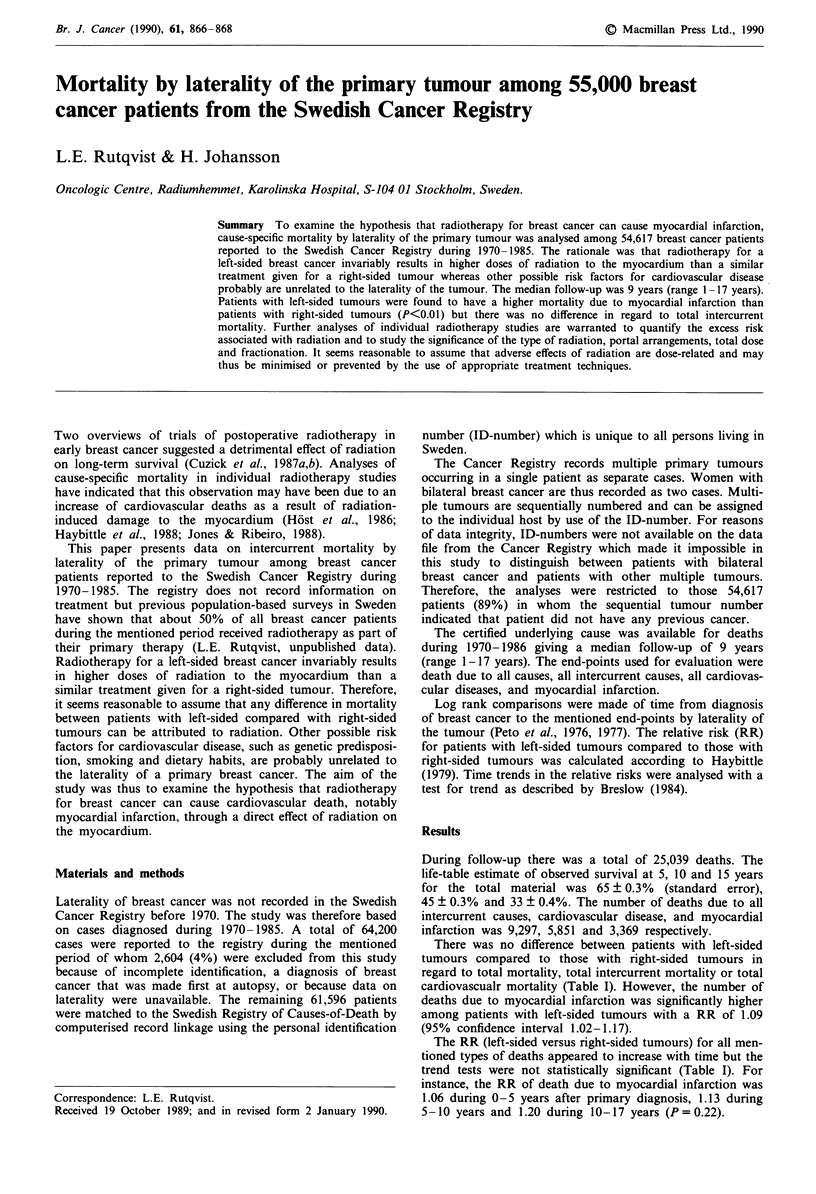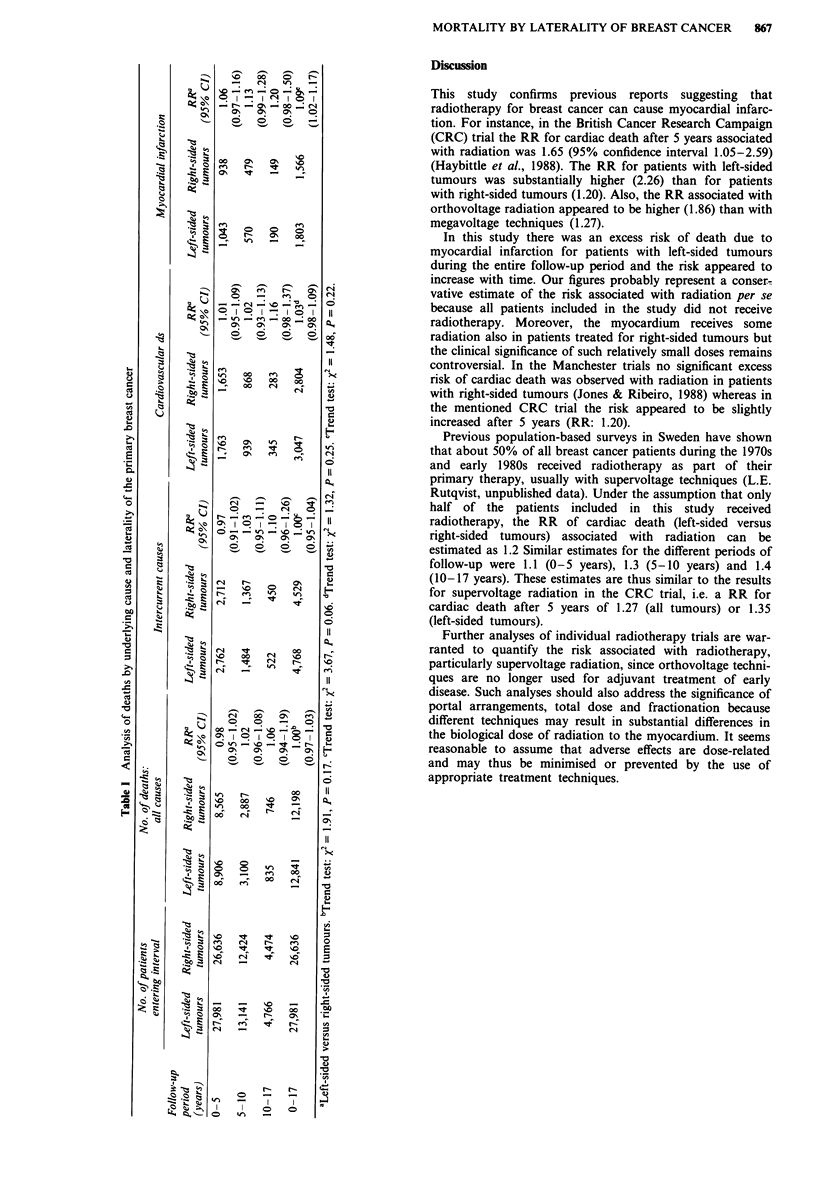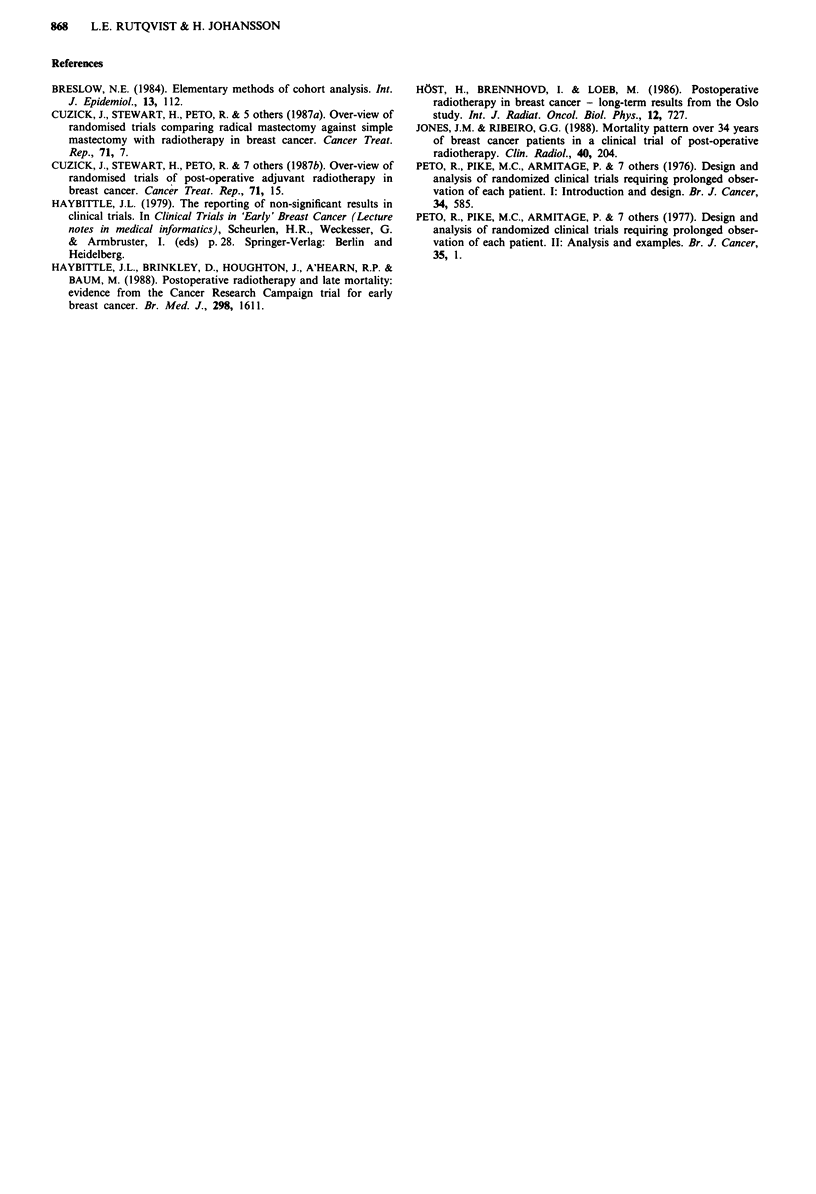# Mortality by laterality of the primary tumour among 55,000 breast cancer patients from the Swedish Cancer Registry.

**DOI:** 10.1038/bjc.1990.193

**Published:** 1990-06

**Authors:** L. E. Rutqvist, H. Johansson

**Affiliations:** Oncologic Centre, Radiumhemmet, Karolinska Hospital, Stockholm, Sweden.

## Abstract

To examine the hypothesis that radiotherapy for breast cancer can cause myocardial infarction, cause-specific mortality by laterality of the primary tumour was analysed among 54,617 breast cancer patients reported to the Swedish Cancer Registry during 1970-1985. The rationale was that radiotherapy for a left-sided breast cancer invariably results in higher doses of radiation to the myocardium than a similar treatment given for a right-sided tumour whereas other possible risk factors for cardiovascular disease probably are unrelated to the laterality of the tumour. The median follow-up was 9 years (range 1-17 years). Patients with left-sided tumours were found to have a higher mortality due to myocardial infarction than patients with right-sided tumours (P less than 0.01) but there was no difference in regard to total intercurrent mortality. Further analyses of individual radiotherapy studies are warranted to quantify the excess risk associated with radiation and to study the significance of the type of radiation, portal arrangements, total dose and fractionation. It seems reasonable to assume that adverse effects of radiation are dose-related and may thus be minimised or prevented by the use of appropriate treatment techniques.


Br. J. Cancer (1990), 61, 866-868 © Macmillan Press Ltd., 1990

### Mortality by laterality of the primary tumour among 55,000 breast cancer patients from the Swedish Cancer Registry

L.E. Rutqvist & H. Johansson

*Oncologic Centre, Radiumhemmet, Karolinska Hospital, S-104 01 Stockholm, Sweden.*

**Summary** To examine the hypothesis that radiotherapy for breast cancer can cause myocardial infarction, cause-specific mortality by laterality of the primary tumour was analysed among 54,617 breast cancer patients reported to the Swedish Cancer Registry during 1970-1985. The rationale was that radiotherapy for a left-sided breast cancer invariably results in higher doses of radiation to the myocardium than a similar treatment given for a right-sided tumour whereas other possible risk factors for cardiovascular disease probably are unrelated to the laterality of the tumour. The median follow-up was 9 years (range 1-17 years). Patients with left-sided tumours were found to have a higher mortality due to myocardial infarction than patients with right-sided tumours ($P<0.01$) but there was no difference in regard to total intercurrent mortality. Further analyses of individual radiotherapy studies are warranted to quantify the excess risk associated with radiation and to study the significance of the type of radiation, portal arrangements, total dose and fractionation. It seems reasonable to assume that adverse effects of radiation are dose-related and may thus be minimised or prevented by the use of appropriate treatment techniques.

Two overviews of trials of postoperative radiotherapy in early breast cancer suggested a detrimental effect of radiation on long-term survival (Cuzick *et al.*, 1987*a,b*). Analyses of cause-specific mortality in individual radiotherapy studies have indicated that this observation may have been due to an increase of cardiovascular deaths as a result of radiation-induced damage to the myocardium (Höst *et al.*, 1986; Haybittle *et al.*, 1988; Jones & Ribeiro, 1988).

This paper presents data on intercurrent mortality by laterality of the primary tumour among breast cancer patients reported to the Swedish Cancer Registry during 1970-1985. The registry does not record information on treatment but previous population-based surveys in Sweden have shown that about 50% of all breast cancer patients during the mentioned period received radiotherapy as part of their primary therapy (L.E. Rutqvist, unpublished data). Radiotherapy for a left-sided breast cancer invariably results in higher doses of radiation to the myocardium than a similar treatment given for a right-sided tumour. Therefore, it seems reasonable to assume that any difference in mortality between patients with left-sided compared with right-sided tumours can be attributed to radiation. Other possible risk factors for cardiovascular disease, such as genetic predisposition, smoking and dietary habits, are probably unrelated to the laterality of a primary breast cancer. The aim of the study was thus to examine the hypothesis that radiotherapy for breast cancer can cause cardiovascular death, notably myocardial infarction, through a direct effect of radiation on the myocardium.

### Materials and methods

Laterality of breast cancer was not recorded in the Swedish Cancer Registry before 1970. The study was therefore based on cases diagnosed during 1970-1985. A total of 64,200 cases were reported to the registry during the mentioned period of whom 2,604 (4%) were excluded from this study because of incomplete identification, a diagnosis of breast cancer that was made first at autopsy, or because data on laterality were unavailable. The remaining 61,596 patients were matched to the Swedish Registry of Causes-of-Death by computerised record linkage using the personal identification number (ID-number) which is unique to all persons living in Sweden.

The Cancer Registry records multiple primary tumours occurring in a single patient as separate cases. Women with bilateral breast cancer are thus recorded as two cases. Multiple tumours are sequentially numbered and can be assigned to the individual host by use of the ID-number. For reasons of data integrity, ID-numbers were not available on the data file from the Cancer Registry which made it impossible in this study to distinguish between patients with bilateral breast cancer and patients with other multiple tumours. Therefore, the analyses were restricted to those 54,617 patients (89%) in whom the sequential tumour number indicated that patient did not have any previous cancer.

The certified underlying cause was available for deaths during 1970-1986 giving a median follow-up of 9 years (range 1-17 years). The end-points used for evaluation were death due to all causes, all intercurrent causes, all cardiovascular diseases, and myocardial infarction.

Log rank comparisons were made of time from diagnosis of breast cancer to the mentioned end-points by laterality of the tumour (Peto *et al.*, 1976, 1977). The relative risk (RR) for patients with left-sided tumours compared to those with right-sided tumours was calculated according to Haybittle (1979). Time trends in the relative risks were analysed with a test for trend as described by Breslow (1984).

### Results

During follow-up there was a total of 25,039 deaths. The life-table estimate of observed survival at 5, 10 and 15 years for the total material was $65 \pm 0.3\%$ (standard error), $45 \pm 0.3\%$ and $33 \pm 0.4\%$. The number of deaths due to all intercurrent causes, cardiovascular disease, and myocardial infarction was 9,297, 5,851 and 3,369 respectively.

There was no difference between patients with left-sided tumours compared to those with right-sided tumours in regard to total mortality, total intercurrent mortality or total cardiovascualr mortality (Table I). However, the number of deaths due to myocardial infarction was significantly higher among patients with left-sided tumours with a RR of 1.09 (95% confidence interval 1.02-1.17).

The RR (left-sided versus right-sided tumours) for all mentioned types of deaths appeared to increase with time but the trend tests were not statistically significant (Table I). For instance, the RR of death due to myocardial infarction was 1.06 during 0-5 years after primary diagnosis, 1.13 during 5-10 years and 1.20 during 10-17 years ($P = 0.22$).

Correspondence: L.E. Rutqvist.
Received 19 October 1989; and in revised form 2 January 1990.

**Table I** Analysis of deaths by underlying cause and laterality of the primary breast cancer

| Follow-up period (years) | No. of patients entering interval: Left-sided tumours | Right-sided tumours | No. of deaths: all causes: Left-sided tumours | Right-sided tumours | RR[a] (95% CI) | Intercurrent causes: Left-sided tumours | Right-sided tumours | RR[a] (95% CI) | Cardiovascular ds: Left-sided tumours | Right-sided tumours | RR[a] (95% CI) | Myocardial infarction: Left-sided tumours | Right-sided tumours | RR[a] (95% CI) |
|---|---|---|---|---|---|---|---|---|---|---|---|---|---|---|
| 0–5 | 27,981 | 26,636 | 8,906 | 8,565 | 0.98 (0.95–1.02) | 2,762 | 2,712 | 0.97 (0.91–1.02) | 1,763 | 1,653 | 1.01 (0.95–1.09) | 1,043 | 938 | 1.06 (0.97–1.16) |
| 5–10 | 13,141 | 12,424 | 3,100 | 2,887 | 1.02 (0.96–1.08) | 1,484 | 1,367 | 1.03 (0.95–1.11) | 939 | 868 | 1.02 (0.93–1.13) | 570 | 479 | 1.13 (0.99–1.28) |
| 10–17 | 4,766 | 4,474 | 835 | 746 | 1.06 (0.94–1.19) | 522 | 450 | 1.10 (0.96–1.26) | 345 | 283 | 1.16 (0.98–1.37) | 190 | 149 | 1.20 (0.98–1.50) |
| 0–17 | 27,981 | 26,636 | 12,841 | 12,198 | 1.00[b] (0.97–1.03) | 4,768 | 4,529 | 1.00[c] (0.95–1.04) | 3,047 | 2,804 | 1.03[d] (0.98–1.09) | 1,803 | 1,566 | 1.09[e] (1.02–1.17) |

[a]Left-sided versus right-sided tumours. [b]Trend test: $\chi^2 = 1.91$, $P = 0.17$. [c]Trend test: $\chi^2 = 3.67$, $P = 0.06$. [d]Trend test: $\chi^2 = 1.32$, $P = 0.25$. [e]Trend test: $\chi^2 = 1.48$, $P = 0.22$.

### Discussion

This study confirms previous reports suggesting that radiotherapy for breast cancer can cause myocardial infarction. For instance, in the British Cancer Research Campaign (CRC) trial the RR for cardiac death after 5 years associated with radiation was 1.65 (95% confidence interval 1.05–2.59) (Haybittle *et al.*, 1988). The RR for patients with left-sided tumours was substantially higher (2.26) than for patients with right-sided tumours (1.20). Also, the RR associated with orthovoltage radiation appeared to be higher (1.86) than with megavoltage techniques (1.27).

In this study there was an excess risk of death due to myocardial infarction for patients with left-sided tumours during the entire follow-up period and the risk appeared to increase with time. Our figures probably represent a conservative estimate of the risk associated with radiation *per se* because all patients included in the study did not receive radiotherapy. Moreover, the myocardium receives some radiation also in patients treated for right-sided tumours but the clinical significance of such relatively small doses remains controversial. In the Manchester trials no significant excess risk of cardiac death was observed with radiation in patients with right-sided tumours (Jones & Ribeiro, 1988) whereas in the mentioned CRC trial the risk appeared to be slightly increased after 5 years (RR: 1.20).

Previous population-based surveys in Sweden have shown that about 50% of all breast cancer patients during the 1970s and early 1980s received radiotherapy as part of their primary therapy, usually with supervoltage techniques (L.E. Rutqvist, unpublished data). Under the assumption that only half of the patients included in this study received radiotherapy, the RR of cardiac death (left-sided versus right-sided tumours) associated with radiation can be estimated as 1.2 Similar estimates for the different periods of follow-up were 1.1 (0–5 years), 1.3 (5–10 years) and 1.4 (10–17 years). These estimates are thus similar to the results for supervoltage radiation in the CRC trial, i.e. a RR for cardiac death after 5 years of 1.27 (all tumours) or 1.35 (left-sided tumours).

Further analyses of individual radiotherapy trials are warranted to quantify the risk associated with radiotherapy, particularly supervoltage radiation, since orthovoltage techniques are no longer used for adjuvant treatment of early disease. Such analyses should also address the significance of portal arrangements, total dose and fractionation because different techniques may result in substantial differences in the biological dose of radiation to the myocardium. It seems reasonable to assume that adverse effects are dose-related and may thus be minimised or prevented by the use of appropriate treatment techniques.

### References

BRESLOW, N.E. (1984). Elementary methods of cohort analysis. *Int. J. Epidemiol.*, **13**, 112.

CUZICK, J., STEWART, H., PETO, R. & 5 others (1987*a*). Over-view of randomised trials comparing radical mastectomy against simple mastectomy with radiotherapy in breast cancer. *Cancer Treat. Rep.*, **71**, 7.

CUZICK, J., STEWART, H., PETO, R. & 7 others (1987*b*). Over-view of randomised trials of post-operative adjuvant radiotherapy in breast cancer. *Cancer Treat. Rep.*, **71**, 15.

HAYBITTLE, J.L. (1979). The reporting of non-significant results in clinical trials. In *Clinical Trials in 'Early' Breast Cancer (Lecture notes in medical informatics)*, Scheurlen, H.R., Weckesser, G. & Armbruster, I. (eds) p. 28. Springer-Verlag: Berlin and Heidelberg.

HAYBITTLE, J.L., BRINKLEY, D., HOUGHTON, J., A'HEARN, R.P. & BAUM, M. (1988). Postoperative radiotherapy and late mortality: evidence from the Cancer Research Campaign trial for early breast cancer. *Br. Med. J.*, **298**, 1611.

HÖST, H., BRENNHOVD, I. & LOEB, M. (1986). Postoperative radiotherapy in breast cancer – long-term results from the Oslo study. *Int. J. Radiat. Oncol. Biol. Phys.*, **12**, 727.

JONES, J.M. & RIBEIRO, G.G. (1988). Mortality pattern over 34 years of breast cancer patients in a clinical trial of post-operative radiotherapy. *Clin. Radiol.*, **40**, 204.

PETO, R., PIKE, M.C., ARMITAGE, P. & 7 others (1976). Design and analysis of randomized clinical trials requiring prolonged observation of each patient. I: Introduction and design. *Br. J. Cancer*, **34**, 585.

PETO, R., PIKE, M.C., ARMITAGE, P. & 7 others (1977). Design and analysis of randomized clinical trials requiring prolonged observation of each patient. II: Analysis and examples. *Br. J. Cancer*, **35**, 1.